# Editorial: Protein homeostasis in growth, development and disease

**DOI:** 10.3389/fcell.2023.1150158

**Published:** 2023-02-06

**Authors:** Silvia Masciarelli, Francesco Fazi, Linda M. Hendershot

**Affiliations:** ^1^ Department of Anatomical, Histological, Forensic and Orthopedic Sciences, Sapienza University of Rome, Rome, Italy; ^2^ Department of Tumor Cell Biology, St. Jude Children’s Research Hospital, Memphis, TN, United States

**Keywords:** proteostasis, molecular chaperones and chaperonins, stress responses, homeostasis and differentiation, quality control in the endoplasmic reticulum

## Introduction

The transition of a linear polypeptide chain into a functional, 3-dimensional protein is fraught with dangers, but a successful outcome is critical to all aspects of life. To assist and monitor this process, all cells and organism are endowed with a cadre of molecular chaperones, their co-factors and folding enzymes (Balchin et al., 2016). The ability of the cell to rapidly regulate the availability of these chaperones and folding enzymes is necessary to maintain cellular and organellar homeostasis in response to changes in secretory capacity or alterations in the cellular environment that adversely affect protein folding (Richter et al., 2010; Walter and Ron, 2011). Imbalances in these can lead to disease. In addition to these ubiquitously expressed components of protein folding and quality control systems, tissue-specific factors exist that are more restrictive in their clients and are often important in developmental and differentiation processes. This Research Topic is comprised of a combination of original research articles and reviews that address cutting edge work in this field.

### Generalist chaperones

As polypeptide chains emerge from the ribosome, they can begin to fold co-translationally, which could inhibit appropriate interactions with more distal sequences needed to reach their native states. Chaperone families including Hsp70, Hsp90 and the cage-like chaperonins are found in all lineages of organisms (bacteria, archaea, and both plant and animal eukaryotes) and serve to protect reactive regions on nascent proteins. They are particularly non-selective and can recognize a vast number of sequence unrelated proteins and can therefore be considered generalists. Hsp70s recognize linear stretches of amino acids that are hydrophobic in nature and will ultimately be buried upon correct protein folding and/or assembly (Blond-Elguindi et al., 1993). They prevent misfolding by protecting these regions but do not directly contribute to folding. Conversely, the chaperonins are multi-subunit ring structures that provide a protective cavity that accommodates partially folded clients and drives their proper maturation through nucleotide-dependent interactions between the various subunits and the incompletely folded client (Gestaut et al., 2019). One review in this series addresses recent findings that contribute to our understanding of how the mammalian chaperonin TRiC (Tailless complex polypeptide 1 (TCP-1) Ring Complex) is able to contribute to the folding of proteins too large to fit into its central cavity (Ghozlan et al.). Another review focuses on prefoldin, an adapter that conveys clients to TRiC to maintain protein homeostasis in physiological and pathological conditions (Tahmaz et al.). A third review summarizes studies revealing the role of generalist chaperones in protecting the proteome of the parasite and in renovating the host proteome to its own benefit (Blatch).

### Quality control in the endoplasmic reticulum

The endoplasmic reticulum (ER) is the gateway into the secretory pathway and is responsible for the biosynthesis of 1/3 of the proteins encoded in the mammalian genome, which includes proteins that are secreted, expressed on the cell surface, or that populate all organelles of the secretory pathway (Dancourt and Barlowe, 2010). Stringent quality control programs comprised of molecular chaperones and folding enzymes aid and monitor the maturation of nascent proteins entering this organelle (Wiseman et al., 2022). While bound to the chaperone, incompletely folded clients are prevented from moving further along the secretory pathway due to retention sequences on the chaperones. In the case of most soluble chaperones, the tetrapeptide sequence, KDEL, is encoded at their C-terminus. Should the chaperone exit the ER incorrectly it encounters a KDEL receptor in the intermediate compartment or *cis* Golgi, which returns it and any bound client to the ER (Lewis et al., 1990). A review in this Research Topic discusses variations in recycling rates that alter the concentration of individual soluble chaperones in specific proximal regions of the secretory pathway, as well as examples where capture and return fails allowing specific soluble chaperones to be secreted (Palazzo et al.). Equally important to maintaining the secretory proteome is the ability to identify proteins that ultimately fail to mature and/or assemble properly and target them for degradation. In most cases this involves extracting the protein from the ER for degradation by cytosolically localized proteasomes in a process referred to as ER-associated degradation (ERAD) (Vembar and Brodsky, 2008; Hwang and Qi, 2018). The presence of a single unfolded domain or region necessitates that the protein be degraded raising the question of how folded regions are accommodated for passage through an intermembrane channel. An original research study identifies the checkpoints in this process and the components that execute them (Mann et al.).

### Regulating chaperone levels to accommodate secretory loads during differentiation

An ER stress-sensing response termed the Unfolded Protein Response (UPR) transcriptionally adjusts chaperone levels to the secretory capacity of the cell (Walter and Ron, 2011). As such the UPR provides an essential function during the differentiation of highly secretory cells like plasma cells (Iwakoshi et al., 2003), and in response to environmental cues received by insulin-secreting β islet cells (Scheuner et al., 2005) to enhance and populate the secretory pathway. An original research paper in this Research Topic explores temporal changes in the proteostasis network and their triggers, which provide massive reshaping of the secretory pathway during the progesterone-stimulated differentiation of endometrial stromal cells allowing allows them to secrete large quantities of factors necessary for embryo implantation (Pittari et al.).

### Tissue-specific chaperones and differentiation

The chaperone system is extremely plastic. For example, generalist chaperones, like those of the Hsp70 family, can cooperate with different co-chaperones, changing their substrate specificity (Kampinga and Craig, 2010). Higher eukaryotes, composed of many different cell types, tissues, and organs, present different folding demands that must be specifically addressed. A recent systematic analysis of the basal chaperone network in a variety of human tissues experiencing normal physiological conditions showed that most chaperones have tissue-specific behaviors, and this organization is established during organ development, maintained through adulthood, and altered in aging (Shemesh et al., 2021). The same research group contributed to this Research Topic with two original papers focusing on tissue-specific regulation of chaperone expression. In one they identified a neural G protein coupled receptor as a key regulator of proteostasis in different somatic tissues, which is required to transfer resources to progeny production during the transition to adulthood in *Caenorhabditis elegans* (Kishner et al.). In the other they show that the *C. elegans* ortholog of the main muscle transcription factor MyoD, is essential to preserve proper expression of the muscle specific chaperone system not only during differentiation but also in adulthood (Nisaa and Ben-Zvi).

### Chemical manipulation of proteostasis for therapeutic interventions

Whereas the core proteostasis network consists of the mechanisms driving protein translation, folding and degradation, stress response pathways such as the UPR, the Heat Shock Response (HSR), the Integrated Stress Response (ISR) and the oxidative stress response add levels of regulation that allow the cell to cope with and survive situations that unbalance proteostasis (Brehme et al., 2019). Cancer cells are able to survive under prolonged conditions of proteotoxic stress that is due to an unfavorable environment, including hypoxic and insufficient nutrients, by activating pro-survival stress responses. When these mechanisms of adaptation are not sufficient to handle proteostasis alterations, cells undergo apoptosis. Thus, there is a growing interest in targeting proteostasis to induce death of cancer cells (Marciniak et al., 2022; Wolska-Washer and Smolewski, 2022; Śniegocka et al., 2022). In this context, a review article in this Research Topic discusses the potential of using ascorbic acid (Vitamin C) as an adjuvant therapy for acute myeloid leukemia on the basis of its role in epigenetics and of its pro-oxidant properties (Travaglini et al.).
